# Growth and Performance of Perovskite Semiconductor CsPbX_3_ (X = Cl, Br, I, or Mixed Halide) for Detection and Imaging Applications

**DOI:** 10.3390/ma17215360

**Published:** 2024-11-01

**Authors:** R. Hawrami, L. Matei, E. Ariesanti, V. Buliga, H. Parkhe, A. Burger, J. Stewart, A. Piro, F. De Figueiredo, A. Kargar, K. S. Bayikadi, J. Reiss, D. E. Wolfe

**Affiliations:** 1 Department of Life and Physical Sciences, Physics Division, Fisk University, 1000 17th Avenue N, Nashville, TN 37208, USA; 2 Radiation Monitoring Devices, Inc., 44 Hunt Street, Watertown, MA 02472, USA; 3 Department of Chemistry, Northwestern University, 2145 Sheridan Road, Evanston, IL 60208, USA; 4 Department of Materials Science and Engineering, Pennsylvania State University, University Park, PA 16802, USA

**Keywords:** cesium lead halide perovskite, bulk single crystal growth, melt growth, room temperature semiconductor detector

## Abstract

The material family halide perovskites has been critical in recent room-temperature radiation detection semiconductor research. Cesium lead bromide (CsPbBr_3_) is a halide perovskite that exhibits characteristics of a semiconductor that would be suitable for applications in various fields. In this paper, we report on the correlations between material purification and crystal material properties. Crystal boules of CsPbX_3_ (where X = Cl, Br, I, or mixed) were grown with the Bridgman growth method. We describe in great detail the fabrication techniques used to prepare sample surfaces for contact deposition and sample testing. Current–voltage measurements, UV–Vis and photocurrent spectroscopy, as well as photoluminescence measurements, were carried out for material characterization. Bulk resistivity values of up to 3.0 × 10^9^ Ω∙cm and surface resistivity values of 1.3 × 10^11^ Ω/□ indicate that the material can be used for low-noise semiconductor detector applications. Preliminary radiation detectors were fabricated, and using photocurrent measurements we have estimated a value of the mobility–lifetime product for holes (μτ)_h_ of 2.8 × 10^−5^ cm^2^/V. The results from the sample testing can shed light on ways to improve the crystal properties for future work, not only for CsPbX_3_ but also other halide perovskites.

## 1. Introduction

Finding the next room-temperature semiconductor detector that will transform the future of radiation detection technology has been the pursuit of many researchers around the world. The growing interest in room-temperature radiation detectors has led to an extensive effort to develop new materials, both scintillators and semiconductors, that address specific challenges of various applications in the fields of homeland security (nonproliferation of nuclear materials) and industrial and medical imaging, as well as fundamental scientific research [1,2,3]. Compared to scintillators, which have the intrinsic non-proportionality of the light yield and the obvious need of a photosensor to convert the light into electrical signal, semiconductor radiation detectors present the advantage of direct conversion characteristics while providing a superior spectroscopic performance [4,5]. Semiconductors can be easily integrated in front-end electronics systems to achieve a better performance in terms of energy resolution and spatial resolution, if pixelated contacts are used [6].

There has been extensive research conducted for room-temperature radiation detection semiconductors. Cadmium zinc telluride (CZT) is a room-temperature semiconductor detector which has been substantially researched over the last thirty years. Because CZT is sensitive to a wide range of photon energy, it can be used in many applications. The Bridgman melt growth method has been the main method to produce CZT crystal ingots. This growth method, however, causes many crystalline defects such as twinning, stacking faults, grain boundaries, and zinc segregation [7]. Phase defects are another limitation observed when growing CZT. While CZT has excellent charge properties, these growth and material issues have prevented the widespread use of CZT as a room-temperature detector due to availability and production cost.

Another promising radiation detector material is thallium bromide (TlBr), which has been hailed as the next promising material among wide bandgap semiconductor materials to rival CZT [8]. The high stopping power of its elements (Z_eff_ = 58) and density (7.56 g/cm^3^) make it suitable for many applications in astrophysics, medicine, and military applications [8]. However, TlBr is soft and current material quality limits its gamma ray detection capabilities [8]. Both of these properties make TlBr difficult to develop for widespread use.

Cesium lead bromide CsPbBr_3_ (CPB) is the latest promising room-temperature semiconductor detector that has been the focus of recent research [9,10,11,12,13,14,15,16]. It falls into the halide perovskite family of materials that follows an ABX_3_ crystal structure. CPB is a promising semiconductor crystal in that it has optoelectronic properties characterized by a high carrier mobility, as well as a large bandgap (2.25 eV) which offers the possibility of low noise performance at room temperature. The all-inorganic perovskite CPB, with its wide band and broad valence and conduction bands, can be grown in a large size both from melt and from solution [9,10,15,16]. CPB has a high effective atomic number, *Z*_eff_, of 65.9, which is higher than CZT (*Z*_eff_ = 50.2), leading to higher photon stopping power especially in the high-energy region (>500 keV). The quality of the grown material can be improved by including a zone-refining step after material synthesis, which resulted in a higher purity material being grown. The grown crystal demonstrated the capability to detect alpha particles and gamma radiation. The use of asymmetric contacts on the wafers of CsPbBr_3_ proved to optimize the leakage currents and improve the detection capabilities [11,12,13,14].

In this paper, the growth and the characterization of CPB crystals at Fisk University (Fisk) is described. Pre-growth material purification by zone-refining was conducted in-house, which resulted in pre-cursor materials with a purity of 99.999%. The purified materials were used to grow single bulk CPB ingots by the Bridgman method. While the growth optimization of CPB is the main factor that improves material properties, different fabrication techniques, such as etching with different bromine concentration and different immersion times, also have the potential to improve detector performance. CPB meets most of the requirements for X-ray and gamma ray detection with fewer limitations compared to other room-temperature radiation detector materials.

## 2. Experimental Methods

### 2.1. Material Purification and Crystal Growth

**Figure 1:**
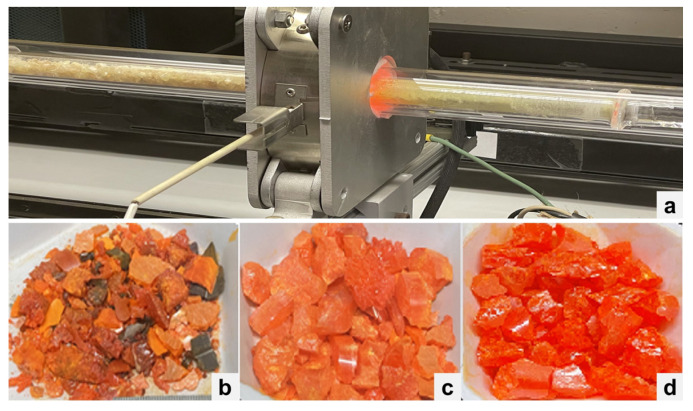
(**a**) Purification of CsPbBr_3_ with a zone-refining system at Fisk. (**b**) CsPbBr_3_ synthesized from the as-received materials. (**c**) Cleaned CsPbBr_3_ synthesized materials. (**d**) Zone-refined CsPbBr_3_ materials.

Crystals with a low defect density are crucial for semiconductor spectrometer performance. Such crystals are usually grown with >5 N precursor materials. The raw starting materials CsX and PbX_2_ (X = Cl, Br, I) with 4–5 N purity levels were ordered from different chemical vendors. The growth process was started by synthesizing CsPbX_3_ with the as-received materials. The synthesized material was then used as a charge for initial growth runs. Purification of the synthesized charge was carried out when the purity level was considered not acceptable, based on the crystal color or appearance, material analysis using a differential scanning calorimeter, and the device performance. The zone-refining technique [17] was used for the purification of each binary compound CsX and PbX_2_, as well as the synthesized CsPbX_3_ compounds, respectively. Figure 1a shows the zone-refining system and ampoule used to purify CsPbBr_3_. Figure 1b shows the CsPbBr_3_ materials synthesized from the materials as received from the vendors, showing the inclusion of possible carbonaceous or organics impurities. Figure 1c shows the cleaned CsPbBr_3_ that was subsequently used as the charge for the zone-refining process. Figure 1d shows the zone-refined CsPbBr_3_ material that was then used as the growth charge. Material colors observed as seen in Figure 1b–d can be a visual indicator to the quality of the starting growth materials for CsPbBr_3_.

**Figure 2:**
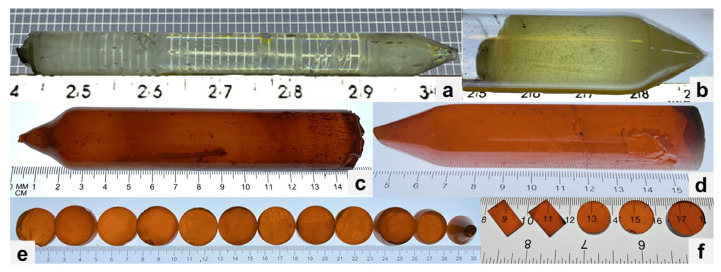
(**a**) CsPbCl_3_ grown with purified materials. (**b**) CsPbCl_3_ grown with non-purified materials. (**c**) CsPbBr_3_ grown with non-purified materials. (**d**) CsPbBr_3_ grown with purified materials. (**e**) CsPbBr_3_ wafers cut transversely. (**f**) CsPbBr_3_ samples cut longitudinally and transversely.

The Bridgman method [18] was used to grow bulk CsPbX_3_ single crystal ingots at Fisk. The purified materials were loaded into a clean quartz ampoule inside an inert atmosphere glove box to grow 15 mm through 51 mm diameter bulk ingots. The ampoule was then attached to a vacuum-heater system, dehydrated, and sealed under high vacuum. The sealed ampoule was loaded into a two-zone vertical furnace with a growth speed of 3.5 mm/h. After crystallization, the furnace was cooled down to room temperature at a rate of 100–150 °C/day. Figure 2a shows an as-grown 16 mm diameter CsPbCl_3_ crystal boule grown with zone-refined starting materials, while Figure 2b shows an as-grown 1-inch diameter CsPbCl_3_ crystal boule grown with non-purified materials. Figure 2c show a 1-inch diameter CsPbBr_3_ crystal boule grown with non-purified materials, while Figure 2d show a 1-inch diameter CsPbBr_3_ crystal boule grown with purified materials. As seen in Figure 2a–d, the CsPbX_3_ ingots grown with purified materials were clear and transparent (Figure 2a,d) compared to the ones grown with non-purified materials (Figure 2b,c).

### 2.2. Crystal Processing, Detector Fabrication, and Characterization Methods

**Figure 3:**
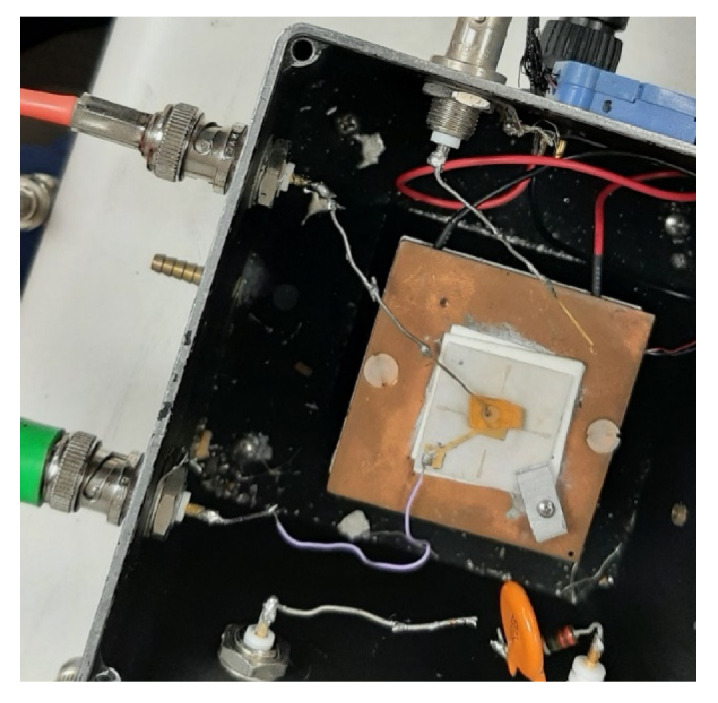
The test box for I–V characteristics.

After growth, the crystal boule was removed from the ampoule and then cut with a diamond wire saw. The crystal boule was cut either longitudinally (along the axis of the boule) or transversely (crosswise). Figure 2e shows CsPbBr_3_ crystal samples cut transversely off the boule, while Figure 2f shows rectangular crystal samples cut longitudinally (along the boule) and circular platelet samples from the boule cut transversely. The cut samples were then lapped (roughly polished) and finely polished with Al_2_O_3_ or SiC polishing papers. Prior to contact deposition, samples were cleaned for less than 10 s in hexane and dried with lint-free wipes. Metal contacts were deposited using RF sputtering. The polished crystals and fabricated devices underwent characterization by several methods. Optical microscope was used to determine cutting direction and crystal quality. Transmission/absorption measurements of polished crystal samples were performed using Agilent Cary 4000 UV–VIS. Current–voltage (I–V) measurements were conducted using a test box fabricated in-house (Figure 3) and a Keithley 237 High Voltage Source Meter unit. Photocurrent spectroscopy was carried out using the same test box now covered by a lid with an aperture to allow light from an Oriel (Newport) 100 W quartz tungsten halogen lamp to reach the sample. Monochromatic light was produced using an Oriel (Newport) C130 monochromator, with the wavelength varied from 200 to 1000 nm in steps of 5 nm. During this variation, the voltage across the sample was maintained at a constant bias. The mobility–lifetime (μτ) product was estimated using photocurrent spectroscopy and alpha particle spectroscopy.

**Figure 4:**
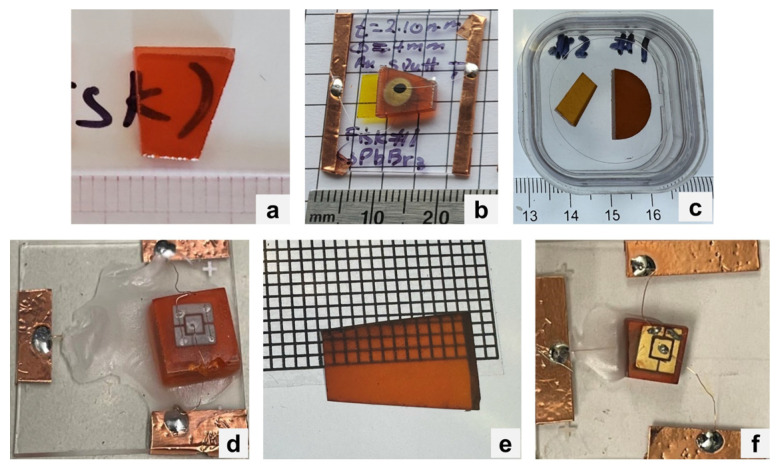
(**a**) CsPbBr_3_ sample sent to RMD. (**b**) Planar device fabricated from the sample in (**a**). (**c**) CsPbBr_3_ samples sent to Penn State. (**d**) CsPbBr_3_ sample fabricated into a device at Northwestern. (**e**) CsPb(Br,I)_3_ sample sent to Northwestern. (**f**) CsPb(Br,I)_3_ device fabricated from the sample in (**e**).

Several CsPbX_3_ crystal samples were sent to several collaborators as bare crystals or with metal contacts. At RMD, Inc. (RMD), CsPbBr_3_ samples (one of them is shown in Figure 4a, with a thickness of 2.1 mm) were fabricated with approximately 600 Å gold (Au) contacts on one side of the samples, while eutectic gallium–indium (GaIn) was used on the opposite side to form rectifying contacts. Carbon paste was used to attach 2-mm palladium (Pd) wires to the Au contacts. Samples were then mounted on glass substrates for characterization. One of the fabricated planar detectors is shown in Figure 4b with the Au contacts facing up. On the fabricated devices, two main measurements were employed for characterization purposes: current–voltage (I–V) measurements and radiation measurements (gamma rays). The planar CsPbBr_3_ detectors were first characterized for current–voltage (I–V) characteristic measurement using a pico-amp meter. Since the mobility–lifetime product of holes is known to be higher than electrons in CsPbBr_3_ [9], the reading electrode should be sensing the holes; therefore, the reading Au contact was grounded (through the preamplifier) and the GaIn contact was positively biased. The wired detector was secured on a glass substrate using Kapton tapes with the Au contact facing up. An aluminum test box was used to test the CsPbBr_3_ planar device for the radiation response. A high-voltage power supply, Cremat CR-110 preamplifier, shaping amplifier, and multi-channel analyzer, and a Tail Pulse Generator (TPG) were used to acquire pulse height spectra. Northwestern University (Northwestern) received a CsPbBr_3_ sample that was fabricated into a device shown in Figure 4c with a 300 nm In as the anode and 150 nm platinum (Pt) as the cathode. Northwestern also received a CsPb(Br,I)_3_ sample (Figure 4d), which was fabricated into a device with a 600 nm sputtered lead (Pb) as the anode and 150 nm evaporated Au as the cathode (Figure 4e).

**Figure 5:**
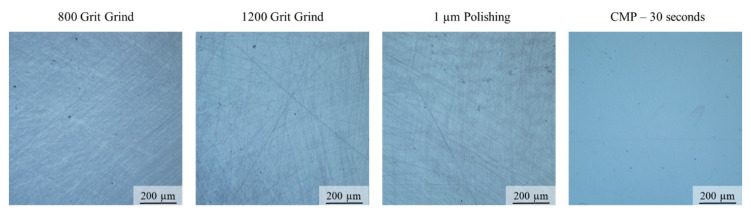
Optical microscopy of a single CsPbBr_3_ crystal taken at each stage of the polishing process. The CMP step significantly reduces observed scratching across the surface of the crystal.

Pennsylvania State University (Penn State) received two CsPbBr_3_ samples: one was cut from the boule crosswise (perpendicular to the growth direction) and one cut from the boule longitudinally (parallel to the growth direction). Surface preparation methods need to be developed in order to obtain ultra-smooth, nm-level surface roughness as it enables reduced surface defect concentration, improved charge transport between the semiconductor and electrode, and overall increased sample reproducibility. Recent literature reports suggest that chemical–mechanical polishing (CMP) approaches may be viable for the CsPbBr_3_ crystal system [19]. This approach was adapted at Penn State and implemented on the CsPbBr_3_ crystals received from Fisk. CsPbBr_3_ crystals were first polished using 800 and 1200 grit SiC paper, followed by a 1 µm polishing suspension on a neoprene polishing pad. Chemical–mechanical polishing was then performed to eliminate further scratching. Crystals were mechanically abraded using the neoprene polishing pad and a chemical etchant lubricant comprised of isopropyl alcohol and dimethylsulfoxide (DMSO) suspension (50/50 vol%). Figure 5 shows optical micrographs of the CsPbBr_3_ crystal surface after each step of the polishing process. The CMP process clearly eliminates all major scratches from the surface, leaving behind only some residual minor scratch marks. Critically, this process also only takes 30 s and can be applied more uniformly across sample sets.

Crystals were then dried with nitrogen and placed into storage in vacuum and in the dark. Passivation was performed using a Plasma-Enhanced Atomic Layer Deposition (PE-ALD) system. In total, 10 nm of SiO_2_ was deposited using a tris(dimethylamino)silane (TDMAS, 99+%) precursor source, with alternating static dose cycles with 400 W O_2_ plasma. To finalize the device fabrication, 50 nm-thick titanium and gold electrodes were deposited via electron-beam physical vapor deposition (EB-PVD). X-ray Diffraction (XRD) was performed on across a 2θ range of 20–80 °C (0.01°/step, 2.5 s/step) using Bragg–Brentano geometry. The operating voltage (40 kV), current (45 mA), and source (Cu, 1.5406 Å) were held constant during all measurements. Phase identification and peak fitting was performed using JADE software. Electrical measurements were conducted with a Keithly 4200 source meter under ambient conditions.

## 3. Results and Analysis

### 3.1. I–V Characteristics and Gamma-Ray Measurements

**Figure 6:**
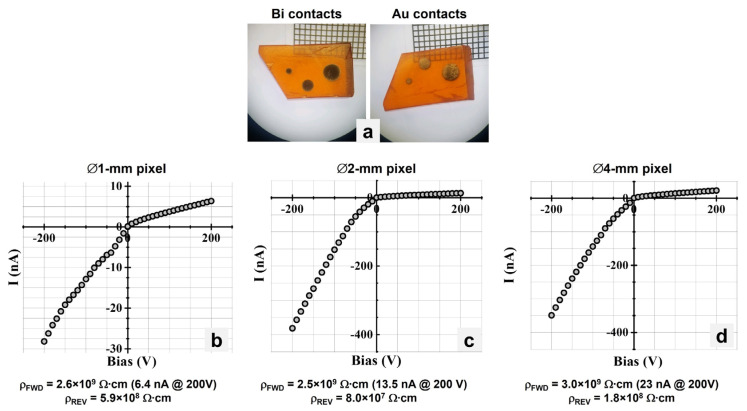
(**a**) CsPbBr_3_ sample with differently sized contacts. (**b**) I–V characteristics for Ø 1 mm contacts. (**c**) I–V characteristics for Ø 2 mm contacts. (**d**) I–V characteristics for Ø 4 mm contacts.

Figure 6a shows a 3 mm-thick CsPbBr_3_ processed sample with three sputtered metal contacts with different diameters: Ø 1 mm, Ø 2 mm, and Ø 4 mm. For this planar device, Au and Bi contacts were deposited using RF sputtering on opposite parallel faces. The sample was placed on a thermo-regulated stage maintained at 20 °C for the duration of the measurements. Variable forward and reverse biases were applied using a source meter and the current was measured for each voltage.

The resistivity was determined using linear least squares fitting (with the formula y=a+bx, where *x* is the applied bias (in V), *y* is the measured current (in Å), *a* is a constant, and *b* is the slope of the line fit) on the I–V characteristics data. The slope of the line fit is the calculated inverse resistance *R*^−1^ (in Ω^−1^). The resistivity *ρ* (in Ω∙cm) is found using the following relationship:

(1) $$R=\rho \frac{L}{A}$$

where *L* is the sample thickness (in cm) and *A* is the contact area (in cm^2^). Figure 6b–d show the I–V characteristic graphs for the differently sized contacts that exhibit rectifying behavior. The bulk resistivity values ranged from 2.2 × 10^9^ to 3.0 × 10^9^ Ω∙cm for forward bias (FWD) and 8.0 × 10^7^ to 5.9 × 10^8^ Ω∙cm for reverse bias (REV). Leakage currents at 6.4 nA, 13.5 nA, and 23 nA were measured at +200 V for Ø 1 mm, Ø 2 mm, and Ø 4 mm contacts, respectively.

**Figure 7:**
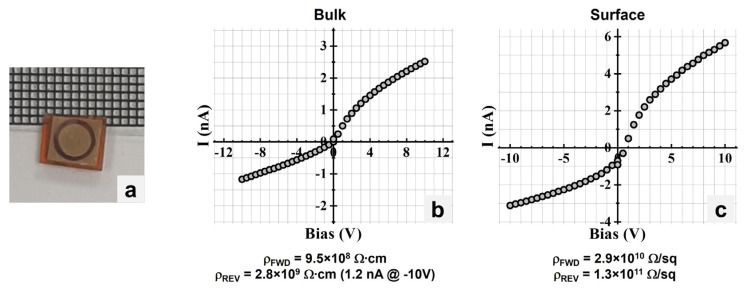
(**a**) CsPbBr_3_ sample with Au–Au contacts and Au guard ring. (**b**) I–V characteristics to determine bulk resistivity. (**c**) I–V characteristics to determine surface resistivity.

Figure 7a shows a 2 mm-thick CsPbBr_3_ sample fabricated with a Au–CsPbBr_3_–Au planar device configuration (Ø 4 mm Au contacts and an Au guard ring with an inner diameter of 5 mm) to determine the bulk and surface resistivities. Figure 7b shows the I–V characteristics to determine the bulk resistivity, while Figure 7c shows the I–V characteristics to determine the surface resistivity. Using Equation (1), the bulk resistivity was calculated to be 9.5 × 10^8^ Ω∙cm for the forward bias (up to +5 V) and 2.8 × 10^9^ Ω∙cm for the reverse bias. A leakage current of 1.2 nA was measured at −10 V. The surface resistivity *ρ_S_* (in Ω/sq) was calculated using the following formula:

(2) $$\rho_{S}=R_{S}\frac{2\pi }{ln\left(\frac{R_{2}}{R_{1}}\right)}$$

where *R_s_* is the surface resistance (in Ω, calculated from the line fit of the I–V characteristics), *R*_1_ (in mm) is the radius of the metal contact, and *R*_2_ (in mm) is the inner diameter of the guard ring. Using Equation (2), the surface resistivity was calculated to be 2.9 × 10^10^ Ω/sq for the forward bias (up to +4.5 V) and 1.3 × 10^11^ Ω/sq for the reverse bias.

**Figure 8:**
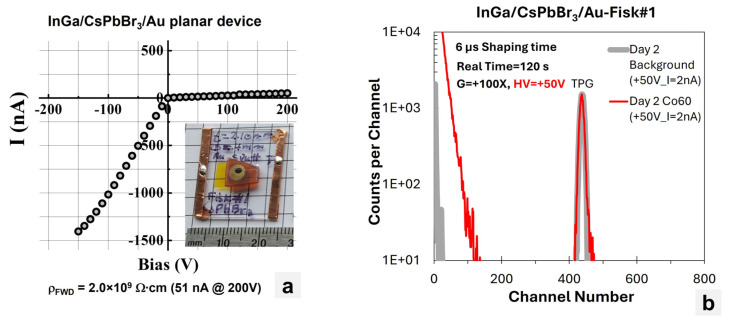
(**a**) I–V characteristics of InGa–CsPbBr_3_–Au planar device (inset) fabricated at RMD. (**b**) Background and ^60^Co spectra collected by the planar device.

Figure 8a shows the I–V characteristics for the CsPbBr_3_ planar device fabricated at RMD (inset picture and Figure 4b). The device showed an effective resistivity of 2 × 10^9^ Ω∙cm for the forward bias and a leakage current of 51 nA at +200 V. Note that due to the different metals being used to form the contacts, a rectifying behavior was observed for the device. Figure 8b shows the background (no source) and ^60^Co spectra collected by the CsPbBr_3_ planar device after two days of conditioning process. The conditioning process refers to a process in which a continuous voltage is applied to a detector for a period of time to improve the device performance. For this measurement, the NIM shaping amplifier was set to a gain of 100 and a shaping time of 6 µs, and the device was irradiated from the top Au contact using a ^60^Co source. The device was initially biased at 50 V for the first day and the bias was increased to 100 V for the second day. The device was unstable and noisy within the first day of conditioning at +50 V and continued to become more stable with time under bias; however, it became noisier and more unstable after the fourth day. Despite the noise level, the leakage current remained acceptably low and stable.

**Figure 9:**
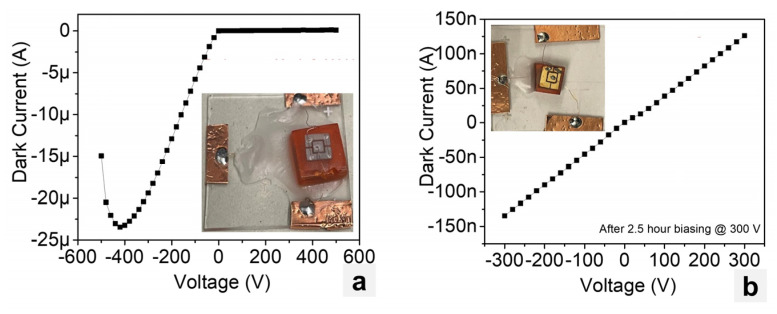
The I–V characteristics for planar devices fabricated at Northwestern: (**a**) In–CsPbBr_3_–Pt and (**b**) Pb–CsPb(Br,I)_3_–Au.

Figure 9a,b show the I–V characteristics for the CsPbBr_3_ and CsPb(Br,I)_3_ planar devices, both fabricated at Northwestern (Figure 4c,e). The In–CsPbBr_3_–Pt exhibited a rectifying behavior with a breakdown voltage around −400 V, while the Pb–CsPb(Br,I)_3_–Au device exhibited a resistive behavior. Similar to the devices fabricated at RMD, the CsPbBr_3_ device fabricated at Northwestern was very noisy; however, the leakage or dark current remained low.

**Figure 10:**
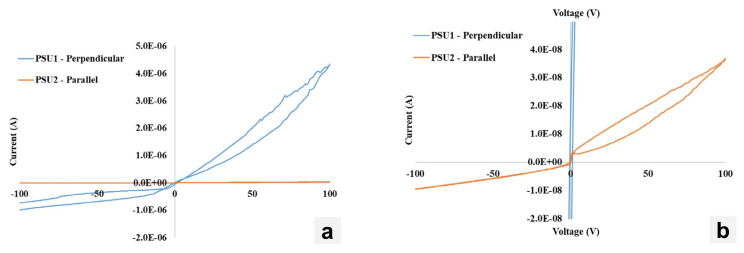
(**a**) I–V characteristics of single CsPbBr_3_ crystals cut perpendicular and parallel to the growth direction. (**b**) Zoomed-in view to resolve the parallel growth direction I–V characteristics.

Figure 10 shows the I–V characteristics of the fabricated CsPbBr_3_ devices with crystals cut perpendicular and parallel to the growth direction. The perpendicular sample clearly shows an I–V characteristic matching that of a rectifying junction, with a current significantly higher under forward bias than under reverse bias. The magnitude of the measured current is quite high, with µA level currents measured at −100 V. This is two orders of magnitude higher than what is typically expected from CsPbBr_3_. There also appears to be substantial ionic mobility measured, as seen by the opening of the hysteresis in both the forward and reverse bias. Conversely, the CsPbBr_3_ crystal cut parallel to the growth direction features I–V characteristics that are much more promising. Similar to previous investigations, 10 nA dark currents are measured at −100 V. A rectifying junction appears to have formed, with a forward bias ~4× higher than the reverse bias, though this I–V characteristic does not have a clear knee voltage to measure a Schottky barrier height, rather a sharp increase in the measured current at ~0 V. This may be due to the presence of the SiO_2_ layer which may build up charge and discharge over the course of the measurement. Forward bias appears to have some degree of ionic mobility with the presence of a hysteresis loop; however, reverse bias conditions eliminate this with no hysteresis observed. This may be due to which ions are mobile in this crystal system, with negatively charged ions (Br^−^) being more mobile and able to drift under positive bias or may be associated with the electrode/passivation structure being more effective at blocking the collection of ionic-induced current measurements. Through comparing the I–V characteristics of these crystal structures, it is clear that cutting the CsPbBr_3_ parallel to the growth direction is advantageous for lowering measured dark currents.

**Figure 11:**
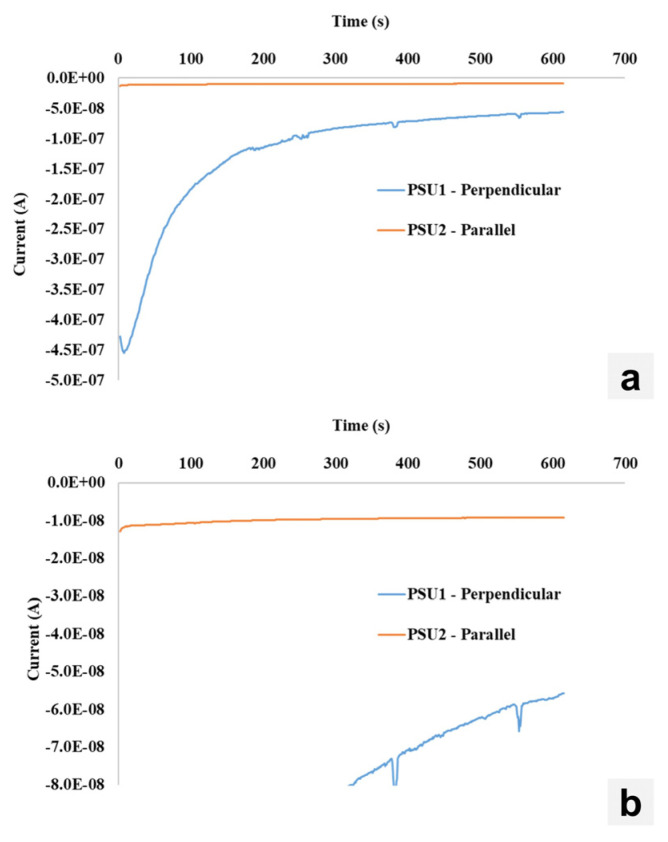
(**a**) Current–time characteristics measured at a constant −100 V bias for CsPbBr_3_ samples cut perpendicular and parallel to the growth direction. (**b**) Zoomed-in view to resolve the parallel direction.

Current–time characteristics were measured to observe the variance of dark current over time. These measurements were conducted at a constant bias of −100 V for 10 min. The sample cut perpendicular to the growth direction begins with a fairly high measured dark current (~−450 nA), which then asymptotically decreases over the measurement period, and begins to level out (~50 nA). This is in stark comparison to the CsPbBr_3_ crystal cut parallel to the growth direction, which has almost no change in the measured dark current throughout the duration of the measurement. In this study, both CsPbBr_3_ crystals were coated with 10 nm of SiO_2_ with identical contact structures, meaning that the degree of surface ion mobility is likely similar between the two crystals. As such, the change in current–time characteristics must be associated with the bulk of the crystal. Recall that the perpendicular-cut sample has more crystallographic boundaries within the bulk, particularly twin boundaries. These boundaries likely serve as fast conduction pathways for both electrons/holes and ions. This is observed under the standard I–V characteristics, where both large dark currents and hysteresis are observed. In the current–time characteristics, changes in the measured current are likely associated with varying ionic contributions during the measurement. During the 10-min measurement window, ions are successfully depleted from the bulk resulting in a lowering and stabilizing dark current measurement. Comparing this to the parallel-cut sample, there are not sufficient pathways (twin/orientation boundaries) to promote bulk ion diffusion. This, combined with the SiO_2_ coating which suppresses surface ion movement, results in a current–time characteristic which is not strongly influenced by moving ions and stays constant throughout (Figure 11). Clearly, crystallographic orientation has a significant effect on both the conductivity of the device and the temporal stability.

Table 1 shows a summary of the CsPbBr_3_ bulk resistivity values measured with different planar device configurations.

### 3.2. Photocurrent Spectroscopy and Optical Measurements

**Figure 12:**
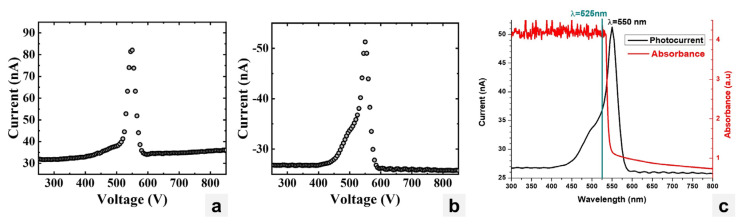
(**a**) Photocurrent spectrum for CsPbBr_3_ at +300 V. (**b**) Photocurrent spectrum for CsPbBr_3_ at −300 V. (**c**) Absorbance spectrum of CsPbBr_3_ with 525 nm light illumination.

Figure 12a shows the photocurrent spectrum of the CsPbBr_3_ sample pictured in Figure 7a held at a constant bias of +300 V, while Figure 12b shows the photocurrent spectrum at −300 V. Both spectra show a peak at 550 nm or 2.25 eV, which is within the bandgap energy range for CsPbBr_3_. In order to estimate the mobility–lifetime product (μτ), the sample was illuminated with a beam of light of 525 nm wavelength that was just below the wavelength of the peak of the photocurrents. At this wavelength, the light was highly absorbed by the sample close to the surface of illumination, and all of the charge carriers generated could travel through the entire thickness of the sample. The choice of the wavelength value is based on the absorption spectrum and photocurrent spectra as shown in Figure 12c.

**Figure 13:**
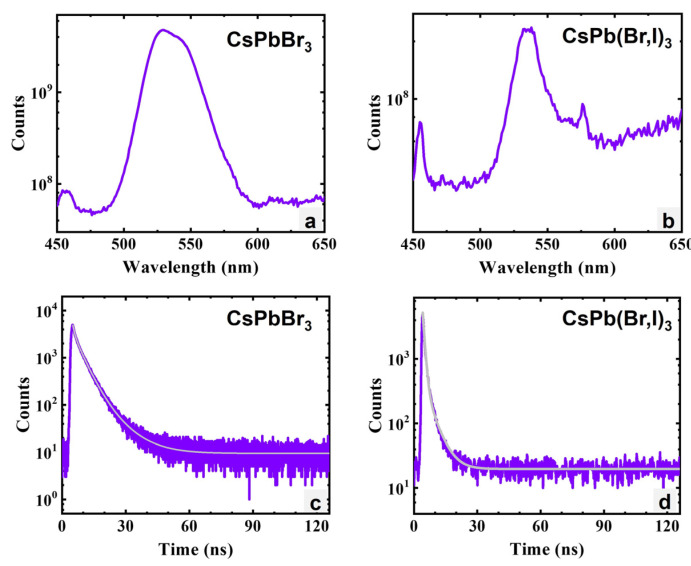
(**a**) Photoluminescence spectrum of CsPbBr_3_. (**b**) Photoluminescence spectrum of CsPb(Br,I)_3_. (**c**) PL lifetime of CsPbBr_3_. (**d**) PL decay curve of CsPb(Br,I)_3_.

Figure 13 shows the photoluminescence and photoluminescence (PL) lifetime measurements on the CsPbBr_3_ and CsPb(Br,I)_3_ devices fabricated at Northwestern. The photoluminescence spectrum of CsPbBr_3_ in Figure 13a shows a broad spectrum with a peak around 529 nm or 2.34 eV, while the photoluminescence spectrum of CsPb(Br,I)_3_ in Figure 13b had a peak around 536 nm or 2.31 eV. The PL lifetime for CsPbBr_3_ was measured at 6 ns (Figure 13c), while the lifetime for CsPb(Br,I)_3_ was measured at 2 ns (Figure 13d). The low lifetime values, which are about 1 to 2 orders of magnitude lower than expected [20], may be caused by impurities and defects. The broad peaks in the room-temperature photoluminescence spectra are an indication of the complex structure of the exciton levels in this class of materials.

### 3.3. Estimation of the Mobility–Lifetime Product (μτ)

**Figure 14:**
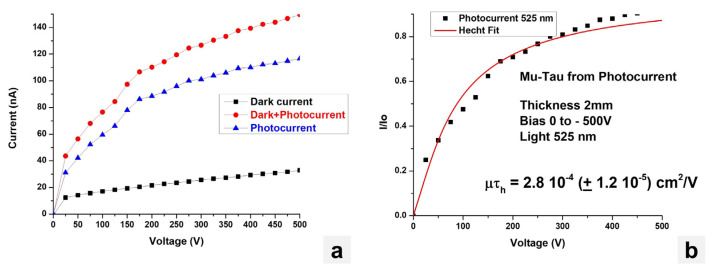
(**a**) Current data measured with and without illumination. (**b**) Photocurrent data fitted with the Hecht equation.

Figure 14a shows the graph of current as a function of applied reverse bias from 0 to −500 V with and without the 525 nm illumination (Figure 12c). The photocurrent curve was obtained by subtracting the dark current (measured without illumination) from the current measured with illumination. The light-generated charge carriers’ contribution was determined by fitting the photocurrent curve using the Hecht equation defined as the following:

(3) $$\eta \left(V\right)=\mu \tau \cdot \frac{V}{L^{2}}\left(1-exp\left(-\frac{L^{2}}{\mu \tau V}\right)\right)$$

where *η* is the charge collection efficiency, *μ* is the mobility (in cm^2^/V∙s), *τ* is the lifetime (in s), *L* is the detector thickness (in cm), and *V* is the applied bias (in volts). Figure 14b shows the photocurrent data fitted using the Hecht equation, resulting in a calculated *μτ* of 2.8 × 10^−4^ cm^2^/V.

**Figure 15:**
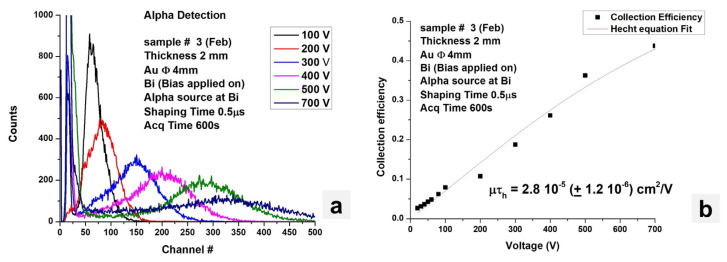
(**a**) Alpha spectra collected by CsPbBr3. (**b**) Peak centroid vs. bias voltage data fitted with Hecht’s equation.

Another commonly used method to determine the *μτ* product is to irradiate the device with alpha particles and determine the peak centroid as the function of the bias voltage. Figure 15a shows the ^241^Am alpha spectra as a function of bias and measured by the CsPbBr_3_ device shown in Figure 7a. Figure 15b shows the graph of the peak centroid as the function of the bias voltage. Fitting the data with the Hecht equation, *μτ* of 2.8 × 10^−5^ cm^2^/V was estimated.

## 4. Conclusions

Using the zone-refining technique, clear and transparent crystal boules were obtained, compared to the unpurified material crystal boules. Crystals up to 1 inch in diameter were grown and cut into wafers with different orientations to evaluate the surface fabrication and contact deposition. Using CMP (chemical–mechanical polishing) techniques, a visibly smooth surface was obtained compared to only using SiC paper. The I–V characteristics showed a low dark current but displayed some noise. Leaving a sample under bias was used to increase the performance, but the sample still displayed noise after too much time under bias. It was observed that cutting the crystal parallel to growth rather than perpendicular resulted in a lower starting dark current. Samples cut perpendicular to crystal growth are believed to have more crystallographic boundaries such as twin boundaries which would explain the discrepancy in the I–V characteristics of the samples. The absorption spectrum of CsPbBr_3_ showed that at 525 nm, light was highly absorbed close to the surface of the sample meaning that the exited charge carrier would travel through the entirety of the sample. We have succeeded in demonstrating the preparation of a high bulk resistivity up to 3.0 × 10^9^ Ω∙cm and wafers with surface resistivity values of 1.3 × 10^11^ Ω/□ which are the pre-requisites in the fabrication of large-volume nuclear radiation detectors. Low lifetime values were also observed during photocurrent decay measurements, which could be a result of impurities and defects in the crystal. The different distinct samples and their respective results will allow improvements to the crystal itself as well as a different approach to the fabrication of samples for future studies and experiments.

## Figures and Tables

**Table 1:** Bulk resistivity values measured with different contact configurations.

Sample Contacts	*ρ* fwd (Ω·cm)	*ρ* rev (Ω·cm)
Bi–Au	2.5–3 × 10^9^	1.8–5.9 × 10^8^
Au–Au	9.5 × 10^8^	2.8 × 10^9^
InGa–Au	2 × 10^9^	
In–Au	2.5 × 10^9^	10^10^

## Data Availability

Contact Corresponding Author for more information.
